# Mindfulness Is Associated with the Metabolic Syndrome among Individuals with a Depressive Symptomatology

**DOI:** 10.3390/nu10020232

**Published:** 2018-02-17

**Authors:** Erika Guyot, Julia Baudry, Serge Hercberg, Pilar Galan, Emmanuelle Kesse-Guyot, Sandrine Péneau

**Affiliations:** 1 Nutritional Epidemiology Research Team (EREN), Centre of Research in Epidemiology and Statistics Sorbonne Paris Cité, Inserm (U1153), Inra (U1125), Cnam, Paris 13 University, COMUE Sorbonne Paris Cité, 93017 Bobigny, France; j.baudry@uren.smbh.univ-paris13.fr (J.B.); s.hercberg@eren.smbh.univ-paris13.fr (S.H.); p.galan@eren.smbh.univ-paris13.fr (P.G.); e.kesse@eren.smbh.univ-paris13.fr (E.K.-G.); s.peneau@eren.smbh.univ-paris13.fr (S.P.); 2 Surveillance and Nutritional Epidemiology Research Unit, Santé Publique France, Paris 13 University, COMUE Sorbonne Paris Cité, 93017 Bobigny, France; 3 Public Health Department, Avicenne Hospital, 93017 Bobigny, France

**Keywords:** mindfulness, metabolic syndrome x, risk factors, depression, epidemiology

## Abstract

The Metabolic Syndrome (MetS) is a major public health burden. Dispositional mindfulness has recently been associated with eating disorders, being overweight, and could therefore be associated with the MetS. We aimed to examine in a cross-sectional design the relationship between mindfulness, the MetS, and its risk factors in a large sample of the adult general population and the influence of depressive symptomatology on this association. Adults participating in the NutriNet-Santé study who had completed the Five Facets Mindfulness Questionnaire and attended a clinical and biological examination were available for inclusion. Multivariable logistic regression models adjusted for socio-demographic and lifestyle factors were performed. A total of 17,490 individuals were included. Among individuals with a depressive symptomatology, those with higher mindfulness were less likely to have a MetS (OR: 0.73, 95% CI: 0.57–0.93), a high waist circumference, a low HDL-cholesterol level and an elevated fasting blood glucose level (all *p* <0.05). In those without depressive symptomatology, individuals with higher mindfulness were less likely to have a high waist circumference (*p* <0.01). In conclusion, higher mindfulness was associated with lower odds of developing a MetS only among individuals with a depressive symptomatology.

## 1. Introduction

The Metabolic Syndrome (MetS) is a combination of risk factors (abdominal obesity, high triglyceride level, low high density lipoprotein (HDL) cholesterol level, high blood pressure and high fasting glucose level), where three abnormal findings out of five, qualify a person for this condition [1]. Individuals with MetS exhibit higher risk of diabetes and cardiovascular diseases [2], which are major public health burdens [3]. In the mid-2000s, 14.6 to 21.1% of French people presented the MetS [4] and the prevalence was expected to increase in the following years [3]. Given the association between smoking [5], sedentary behavior [6], eating behavior [7] and the MetS, many MetS prevention programs have been based on the implementation of actions targeting these modifiable risk factors. Specific psychological characteristics have also been shown to be associated with the MetS. For example, individuals with a Type D personality, (i.e., who have the tendency to experience negative emotional states across time and situations and to inhibit the expression of these emotions) are more likely to have a MetS and an unhealthy lifestyle [8]. High neuroticism, impulsivity and hostility as well as low agreeableness, are also associated with the MetS whereas high conscientiousness is protective [9]. In addition, there is a reciprocal association between anger and the MetS [10].

Dispositional mindfulness is an adaptive psychological trait which is defined as a non-judgmental awareness of the present moment [11]. It has proven its beneficial effects on physical and mental health, including chronic pain [12], cancer treatments [13], addictions [14] and bipolar disorder [15]. Higher overall dispositional mindfulness is also negatively associated with overweight [16]. However, there is to our knowledge no study on the association between dispositional mindfulness, the MetS, and cardiovascular risk factors. A previous study showed that mind-body practices were negatively associated with the body mass index, the level of triglycerides and fasting blood glucose after adjustment for socio-demographic, lifestyle and health factors [17]. Another study has investigated the association between mind body practices as a whole, (i.e., meditation, movement therapy, breathing exercise, relaxation technics or practices) and the MetS and showed their potential beneficial role in improving cardiovascular risk factors after adjustment for sociodemographic and lifestyle factors [18].

In turn, depression is highly prevalent in developed countries and has become a major public health issue [19]. Depression and depressive symptoms, were shown to be negatively related to mindfulness [20] but also positively associated with the MetS, Park et al. [21] and cardiovascular risk factors [22]. Therefore, we hypothesize that depression could possibly modulate the association between mindfulness and MetS and risk factors.

The aim of the present study was to explore in a cross sectional design the relationship between mindfulness (and its subscales) scores, and the MetS (and its components) among adults participating in the NutriNet-Santé study. The potential effect modification by depressive symptomatology was also investigated.

## 2. Materials and Methods

### 2.1. Study Population

This study was conducted as part of the NutriNet-Santé study which is an on-going, web-based, prospective, and observational cohort study launched in France in May 2009 for at least a 10 year follow up. It aims at investigating the association between nutrition and health as well as determinants of dietary behavior and nutritional status in the general French population (internet-using adult volunteers, aged ≥ 18 years old). The rationale, design and methods of the study have been described elsewhere [23]. Briefly, at inclusion and every year of follow up, participants are asked to complete a set of self-administrated web-based questionnaires assessing socioeconomic conditions, demographic, anthropometric, and lifestyle factors, health status, dietary intake and physical activity. In addition, other optional questionnaires related to determinants of eating behaviors, nutritional status, and specific aspects related to health are sent to participants each month. Participants are also invited for a clinical visit in one of the local centers for a clinical and biological examination. All procedures of the NutriNet-Santé study were approved by the International Review Board of the French Institute for Health and Medical Research (IRB Inserm n° 0000388FWA00005831) and the French National Information and Citizen Freedom Committee (CNIL n° 908450 and n° 909216). All procedures related to the clinical examination were approved by the Consultation Committee for the Protection of Participants in Biomedical Research (C09-42 on May 5th 2010) and the CNIL (n° 1460707). All participants provided an electronic informed consent. Further a written consent was requested from participants attending the clinical examination.

### 2.2. Data Collection

#### 2.2.1. Mindfulness

Dispositional mindfulness was assessed in January 2013 using the French version [24] of the Five Facets Mindfulness Questionnaire (FFMQ) [25] which reflects the propensity of being mindful in daily life. This validated questionnaire [24,25] is one of the most recent and the most used to assess mindfulness as a multidimensional latent variable. The FFMQ is composed of 39 items that explores 5 facets of mindfulness: “observing”, “describing”, “acting with awareness”, “non-judging” and “non-reactivity” [25]. The “observing” subscale refers to sensations, cognitions, emotions as well as odors and shapes of our surroundings. “Describing” notice verbalizing internal experiences. “Acting with awareness” reflects the ability to pay attention of what is being done in the present, in contrast of the notion of automatic pilot. “Non-judging” involves how people go through experiences. “Non-reactivity” refers to the tendency to allow thoughts and feelings to come and go without letting them take over. For a better understanding, the word “pattern” of item 31 which is an Anglicism has been replaced by “contrastes” which is the equivalent in French [16]. Each item is measured in a 5-point Likert-type scale ranging from “never or very rarely true” to “very often or always true”. The score of each facet of mindfulness, as well as the overall mindfulness score, were determinate by summing individual item scores. The scores obtained were divided by the appropriate number of items, offering a possible range from 1 to 5. A high score indicates a high degree of mindfulness. In our sample, the FFMQ displayed good internal consistency with an overall Cronbach’s α = 0.89. The subscales Cronbach’s α ranging from 0.75 for the “non-judging subscale to 0.90 for the describing subscale.

#### 2.2.2. MetS and Cardiovascular Risk Factors

Participants were invited to attend one clinical examination in one of the specific health centers of the study located in various French cities, between 2011 and 2014. During the clinical examination, waist circumference defined as the circumference midway between the lower ribs and iliac crests [26], was measured in standing position with an inelastic tape (nearest cm). Systolic and diastolic blood pressures were measured using an automatic sphygmomanometer (Omron HEM-7015IT, Omron, Rosny-sous-Bois, France). They were defined as the mean between the 3 measures taken at 1-min intervals after a 5-min rest. Fasting blood glucose (hexokinase on C 8000 automat, Abbott, Suresnes, France), HDL cholesterol (direct accelerator C8000, Abbott, Suresnes, France) and serum triglycerides (glycerol kinase C8000, Abbott, Suresnes, France) were measured using blood samples collected after at least 6 h of fasting, which were centralized at a single laboratory (INSA, Tour, France). The use of a medication for hypertension, diabetes, or dyslipidemia was assessed through self-administrated web-based questionnaires at inclusion and each year of follow-up.

The presence of MetS was determined according to Joint Interim Statement [1] as having at least three of the following criteria: abdominal obesity (waist circumference ≥94 cm for men and ≥80 cm for women), hypertriglyceridemia (≥150 mg/dL or antihypertriglyceridemia medication), low HDL-cholesterolemia (<40 mg/dL for men and <50 mg/dL for women or dyslipidemia treatment), high blood pressure (systolic blood pressure/diastolic blood pressure ≥130/85 mm Hg or antihypertensive medication) and hyperglycemia (fasting glucose ≥100 mg/dL or antidiabetic medication).

#### 2.2.3. Depressive Symptomatology

Depressive symptomatology was assessed through self-administrated web-based health questionnaires at inclusion and each year of follow-up. Participants were asked to declare their history of depression with the date of diagnosis when appropriate, and to mention their treatments. All incident and prevalent cases were taken into account.

#### 2.2.4. Covariates: Sociodemographic and Lifestyle Data

Sociodemographic and lifestyle characteristics that have been suggested in the literature to be associated with mindfulness, MetS, or the association between these factors, were selected as covariates [27,28]. Information was collected at inclusion and updated at 1-year intervals. The closest available data to mindfulness were used. Collected data were gender, age, education level (primary, secondary, undergraduate, postgraduate), family status (alone without a child, alone with child(ren), living with a partner without a child, living with a partner with child(ren)), smoking status (never, former, current), physical activity, energy intake and the modified French National Nutrition and Health Program Guideline Score (mPNNS-GS). Physical activity was assessed through the French version of the International Physical Activity Questionnaire (IPAQ) [29]. This questionnaire determined the weekly energy expenditure expressed in metabolic equivalent task minutes per week. The physical activity was considered as low when participants practiced under 30 min/d, moderate when they did 30 to 59 min/d, and high when they did at least 60 min/d. Energy [30] and nutrient intakes [31] were determined with at least three 24 h records (2 during the week, 1 during the weekend). Participants declare each food and beverage consumption through validated photograph portion sizes. Under-reporters were identified on the basis of the method proposed by Black [32] and were excluded from the analyses. We used the (mPNNS-GS to estimate overall diet quality. These scores assessed adherence to the French nutritional guidelines using twelve components: eight are referring to food serving recommendations, and four are covering nutrients or food groups whose intake have to be limited. The scores ranged from 0 to 13.5 points. A higher score indicates a better overall diet quality.

### 2.3. Statistical Analysis

Characteristics of included and excluded individuals, as well as individuals with and without depressive symptomatology, were compared using Mann-Whitney U test or Student’s t tests for continuous variables and Pearson χ2 test for categorical variables. Multivariable logistic regression models were used to estimate the strength of the association between overall score of mindfulness, its subscales, and the MetS. Multivariable logistic regression models were also used to assess the association between overall score of mindfulness and each component of the MetS. Covariates and interaction that reached *p* <0.15 in univariate models were retained for inclusion in the multivariable model. Model 1 was adjusted for sex, age, education level and family status. Model 2 was further adjusted for lifestyle characteristics: physical activity and smoking status, and model 3 for dietary intake characteristics: energy intake and mPNNS-GS. Because of a significant interaction of mindfulness with depressive symptomatology (*p* <0.0001), all analyses were stratified by this factor. Missing data for education level, familial status, physical activity and energy intake were handled with multiple imputation by fully conditional specification (5 imputed datasets). A complete case analysis was performed to verify the consistency of the results obtained after multiple imputation.

Sensitivity analyses were performed among individuals reporting past or present use of antidepressant treatments only. 

All tests of significance were two sided and a *p* < 0.05 was considered significant. All statistical analyses were performed using SAS software (version 9.3; SAS Institute Inc., Cary, NC, USA).

## 3. Results

### 3.1. Characteristics of the Sample

**Figure 1:**
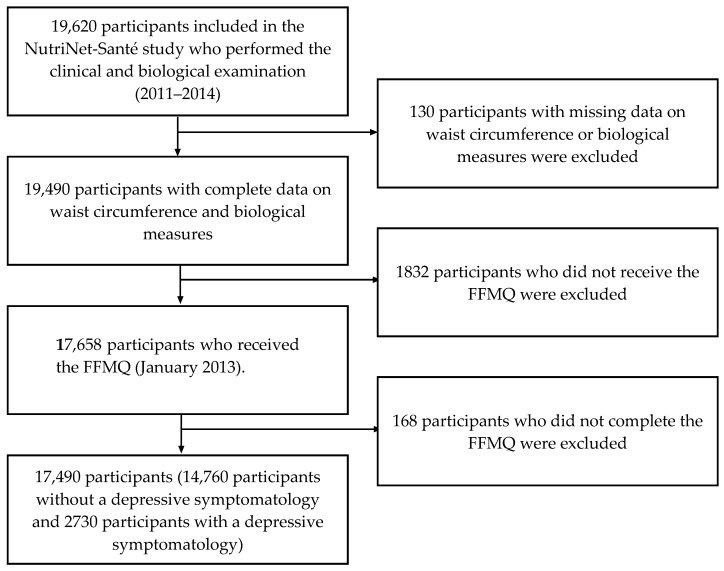
Flow chart for the participants from the NutriNet-Santé cohort study (2011–2014) included in the current analysis.

Of the 19,490 adults who attended the clinical examination and without missing data to assess the presence of the MetS, 17,490 individuals completed the FFMQ and were therefore included in the present analysis (Figure 1). Compared with excluded individuals (those with data for the FFMQ but who did not attend the clinical examination), included participants were older (54.9 ± 13.4 vs. 46.6 ± 14.4 y), less often women (71.8 vs. 80.5%), lived less often with a partner and with child(ren) (17.7 vs. 28.4%), were less often smokers (10.0 vs. 14.4%) and more often highly physically active (39.1 vs. 31.6%), (all *p* < 0.001).

Table 1 shows characteristics of the study sample according to depressive symptomatology. Compared with subjects without a depressive symptomatology, individuals with a depressive symptomatology were more often women, younger, lived more often alone, were less often physically active and more often smokers (all *p* < 0.0001). They had lower scores for overall mindfulness as well as for “acting with awareness”, “non-judging” and “non-reactivity” subscales (all *p* < 0.0001). They were more likely to present MetS, a high waist circumference, a high level of triglycerides and a low level of HDL-cholesterol, or to be treated for these metabolic abnormalities (all *p* < 0.0001). However, they were less likely to have a high blood pressure or to be treated with an antihypertensive treatment (*p* < 0.0001). The average delay between the clinical examination and the FFMQ was 27 ± 18 months in both subgroups (*p* = 0.60).

### 3.2. Association between Mindfulness and Its Subscales and the Metabolic Syndrome According to Depressive Symptomatology

Table 2 shows the association between mindfulness, its subscales, and the MetS, stratified by depressive symptomatology status. Among individuals with a depressive symptomatology, those with higher levels of overall mindfulness were less likely to have a MetS. In addition, individuals with higher levels of “non-judging” and “non-reactivity” were less likely to have a MetS while no significant association was observed for “acting with awareness”, “describing”, and “observing” subscales. Conversely, among individuals without a depressive symptomatology, no association between mindfulness level, its subscales, and the MetS were observed. The three models gave similar results apart for the “observing” subscale, which did not remain significantly associated with the MetS among individuals without a depressive symptomatology in model 2 and 3.

Sensitivity analyses were performed taking into account individuals who reported taking antidepressant treatments only. In this subgroup, the association between mindfulness and the MetS (model 3) was significant (OR: 0.55, 95% CI: 0.37–0.80) (*p* = 0.0018), while it was non-significant among individuals without a depressive symptomatology (OR: 1.00, 95% CI: 0.88–1.13) (*p* = 0.99).

Analyses based on multiple imputation and complete case analysis gave similar results.

### 3.3. Association between Mindfulness and Cardiovascular Risk Factors According to Depressive Symptomatology

Table 3 shows the association between mindfulness, and cardiovascular risk factors, stratified by depressive symptomatology status. Individuals with higher levels of overall mindfulness were less likely to have a high waist circumference independently of depressive symptomatology. In individuals with a depressive symptomatology, those with higher levels of overall mindfulness were less likely to have a low HDL-cholesterol and a high level of fasting blood glucose or a treatment for these metabolic abnormalities, while no significant association was found for blood pressure or triglyceride level. In individuals without a depressive symptomatology, no association was observed for blood pressure, triglycerides, HDL-cholesterol and fasting blood glucose. The three models gave similar results.

## 4. Discussion

The present cross-sectional study showed that individuals with higher levels of overall mindfulness were less likely to present the MetS among individuals with a depressive symptomatology only. This association was particularly observed for the “non-judging” and “non-reactivity” subscales. Overall mindfulness was also associated with waist circumference, HDL-cholesterol and fasting blood glucose risk factors. No association was found between mindfulness and the MetS and cardiovascular risk factors in non-depressive individuals apart from an association between mindfulness and waist circumference.

### 4.1. Mindfulness and the MetS

To our knowledge, no similar study has been conducted so far in the literature. The underlying mechanisms by which mindfulness might affect the MetS are not yet known but are likely to include both physiologic and psychological components.

A first hypothesis includes a mediation of this association by food intake. The MetS has been shown to be associated with unhealthy diet [33] and with specific dietary patterns [27,34,35]. Several studies have indicated that mindfulness encourages healthy diet through energy intake [36]. Dispositional mindfulness has been shown to predict intake of fruits and vegetable and fat [28] and to be an efficient way to reduce effects of hunger on unhealthy food consumption [37]. However, findings on serving size are conflicting [37,38]. In addition, in the present study, further adjustment on dietary quality and total energy intake did not modify the association between mindfulness, the MetS, and its risk factors. These data suggest that the association is not only resulting from a healthy dietary behavior of individuals with a high level of dispositional mindfulness.

Eating disorders might also be a mediator of the association between mindfulness and the MetS. Eating disorders have been shown to be associated with both the MetS [39] and mindfulness [40]. Individuals suffering from eating disorders have defects in emotion processing [41] and mindfulness which might contribute to regulating emotions [42]. In addition, eating disorders have been associated with cognitive rumination [43]. High level of “non-judging” might allow individuals to give free rein to their thoughts without repressing them, which might avoid food-thought suppression. Paradoxically, attempting to avoid unwanted thoughts about eating has been associated with an increase of these thoughts [44] and food-seeking behavior, [45] which might contribute to the development of the MetS. Eating disorders have also been associated with adverse life events [46]. Individuals with higher “non-reactivity” could have a better ability to adapt themselves to these events and not take refuge in food craving since they have a more flexible cognitive control [47].

### 4.2. Mindfulness and Cardiovascular Risk Factors

We found that dispositional mindfulness was inversely associated with waist circumference among all participants (with and without depressive symptomatology). Our results support previous data in the literature showing an inverse association between mindfulness and overweight [16,48].

In our study, higher mindfulness was associated with lower levels of fasting glucose among individuals with a depressive symptomatology. One study showed that individuals with high scores of dispositional mindfulness were more likely to have normal blood glucose level [49], which is consistent with our results. In addition, a randomized control study showed that individuals who received a mindfulness intervention had lower fasting glucose level compared with individuals who did not receive an intervention [50].

In the present study, higher mindfulness was associated with higher levels of HDL-cholesterol only among individuals with a depressive symptomatology but no significant association was found with triglyceridemia. In the literature, one study reported no association between mindfulness and total cholesterol level [48]. Moreover, mindfulness based-intervention did not seem to be effective in the long-term to improve triglyceridemia and triglyceride/HDL ratio [50]. These findings are consistent with our results on individuals without a depressive symptomatology.

We did not find any significant association between mindfulness and blood pressure. Another observational study reported similar results [48]. On the other hand, mindfulness-based interventions have shown to be effective to reduce blood pressure among cardiac patient [51].

Considering all of these elements, mindfulness seems to play a role on the “adiposity”, “HDL” and “fasting glucose” components of the MetS specifically among individual with depressive symptomatology only. The conflicting results regarding the other cardiovascular risk factors suggest that relationship between psychological factors and cardiovascular health are complex and need to be further explored.

### 4.3. Effect Modification by Depressive Symptomatology

The MetS is highly prevalent among individuals with a depressive symptomatology [52]. Especially, major depression predicts the onset of the MetS in middle-aged women [53]. In our study, individuals with a depressive symptomatology had lower mindfulness scores compared with individuals without a depressive symptomatology. Another study showed that mindfulness was negatively associated with depression [20]. In addition, the subscales “acting with awareness” and “non-judging” were found to be associated with lower levels of depressive symptoms [54].

In our study, the association between mindfulness and the MetS was significant only among individuals with a depressive symptomatology. We can hypothesize that dispositional mindfulness might be more useful among individuals with a depressive symptomatology. The use of mindfulness in everyday life could help individuals manage depression and, consequently, the MetS, since they are significantly associated. The positive impact of mindfulness on depression could come from stress management [55]. Stress is associated with the MetS [56], partly due to cortisol release which might lead to an increase in food intake [57].

### 4.4. Strengths and Limitations

An important strength of our study is its large sample size, which provided a high statistical power and allowed stratification on depressive symptomatology status. In addition, important sociodemographic and lifestyle confounding factors have been taken into account. However, we cannot exclude the existence of other potential confounding factors, such as eating disorders, hormones or genetics, in the relationship between mindfulness and the MetS that were not taken into account.

The standardized measurements (waist circumference and biology) are another advantage of the study. The FFMQ is a useful and widespread instrument for measuring mindfulness that has been translated into several languages, including French [24]. It has also been shown to be a reliable and valid questionnaire for assessing mindfulness among depressed individuals [58]. This questionnaire has satisfactory internal consistency, replicated in the present study.

The main limitation of our study is its cross-sectional design which does not allow us to draw a conclusion on causal inference. A reverse causality between the MetS and mindfulness may exist. However, the FFMQ measures dispositional mindfulness, which is a psychological trait expected to be constant over time. Caution is also needed when generalizing our results since the NutriNet-Santé study is a long-term nutrition-focused cohort and participants are recruited on a voluntary basis. Thus, participants are likely to be particularly health conscious and interested in nutritional issues, which may have led to an underestimation of the strength of the association. Finally, individuals included in the present study have different characteristics compared with the whole cohort. For example, individuals who came to the centers to perform the clinical examination were older.

## 5. Conclusions

In our study, higher mindfulness was associated with lower odds of developing a MetS as well as lower waist circumference, higher HDL-cholesterol and a lower level of fasting blood glucose among individuals with a depressive symptomatology. In contrast, the only association observed in individuals without a depressive symptomatology was a negative association between mindfulness and waist circumference. These preliminary findings support the importance to more closely monitor individuals with depressive symptomatology and a low level of mindfulness. Fostering practices that increase mindfulness in programs focusing on the prevention of MetS and related diseases, in particular among subjects with depressive symptomatology which are at risk of cardiovascular disorders, could be of potential interest. More studies, in particular, longitudinal studies using mindfulness-based interventions, are however needed to confirm these findings.

## Figures and Tables

**Table 1:** Socioeconomic and lifestyle characteristics of the participants according to depressive symptomatology in 17,490 individuals (NutriNet-Santé Study, France, 2011–2014) ^1^.

	All *n* = 17,490	No Depressive Symptomatology *n* = 14,760	Depressive Symptomatology *n* = 2730	*P*
Women (%)	71.8	70.0	81.9	
Age (yr)	54.9 ± 13.4	55.1 ± 13.6	53.9 ± 12.4	<0.0001
Education level (%)				0.0028
Primary	2.9	2.9	2.9	
Secondary	31.3	31.1	32.1	
Undergraduate	27.3	27.0	29.4	
Postgraduate	33.6	34.1	31.0	
Missing data	4.8	4.9	4.7	
Family status (%)				<0.0001
Alone without a child	23.8	22.2	32.3	
Alone with child(ren)	3.0	2.7	4.8	
Living with a partner without a child	55.4	57.0	46.4	
Living with a partner with child(ren)	17.6	17.9	16.3	
Missing data	0.2	0.1	0.2	
Physical activity (%)				<0.0001
Low	19.7	18.7	25.0	
Moderate	41.2	41.0	41.9	
High	39.1	40.2	33.1	
Missing data	0.1	0.1	0.0	
Smoking status (%)				<0.0001
Never-smoker	47.5	48.3	43.7	
Former smoker	42.5	42.4	42.8	
Current smoker	10.0	9.4	13.5	
Energy intake (kcal/d) ^2^	1895.8 ± 510.3	1902.7 ± 511.0	1858.6 ± 504.8	<0.0001
Missing data (%)	2.4	2.4	2.5	
mPNNS-GS ^3^	8.02 ± 1.62	8.05 ± 1.61	7.92 ± 1.66	0.0002
Mindfulness scores (1.0–5.0) ^4^	3.3 (1.1–5.0)	3.4 (1.4–5.0)	3.2 (1.1–4.8)	<0.0001
Acting with awareness (1.0–5.0)	3.6 (1.0–5.0)	3.6 (1.0–5.0)	3.4 (1.0–5.0)	<0.0001
Describing (1.0–5.0)	3.3 (1.0–5.0)	3.3 (1.0–5.0)	3.4 (1.0–5.0)	0.85
Observing (1.0–5.0)	3.5 (1.0–5.0)	3.5 (1.0–5.0)	3.5 (1.0–5.0)	0.14
Non-judging (1.0–5.0)	3.5 (1.0–5.0)	3.5 (1.0–5.0)	3.1 (1.0–5.0)	<0.0001
Non-reactivity (1.0–5.0)	2.9 (1.0–5.0)	2.9 (1.0–5.0)	2.7 (1.0–4.9)	<0.0001
Metabolic syndrome (%)	12.3	11.8	15.5	<0.0001
Elevated WC (≥94 cm for men. ≥80 cm for women)	42.8	41.4	50.6	<0.0001
High blood pressure (SBP ≥ 130 and/or DBP ≥ 85 mm Hg) or treatment	41.2	41.8	37.8	<0.0001
High triglycerides (≥150 mg/dL) or treatment	10.2	9.7	13.0	<0.0001
Low HDL-cholesterol (<40 mg/dL for men. <50 mg/dL for women) or treatment	9.9	9.2	13.5	<0.0001
High fasting blood glucose (≥100 mg/dL) or treatment	13.5	13.5	13.3	0.84

^1^ Values are the median (range) or mean ±SD unless noted otherwise. *p* values are for differences between participants with and without a depressive symptomatology on the basis of Mann-Whitney U test, Student’s t-test or Pearson χ2 test as appropriate; ^2^ Including alcohol; 1 kcal/d = 4.18 kJ/d; ^3^ A higher mPNNS-GS score indicates better overall diet quality; ^4^ Score range (higher scores indicated a greater mindfulness).

**Table 2:** Multivariable logistic regression models between mindfulness ^1^ and its subscales ^1^ and the Metabolic Syndrome according to depressive symptomatology in 17,490 individuals (NutriNet-Santé study, France, 2011–2014).

	Models	No Depressive Symptomatology *n* = 14,760		Depressive Symptomatology *n* = 2730	
		OR (95% CI)	*P*	OR (95% CI)	*P*
Overall Mindfulness	Model 1 ^2^	0.96 (0.84–1.09)	0.51	0.71 (0.56–0.91)	0.0072
	Model 2 ^3^	1.01 (0.88–1.15)	0.92	0.72 (0.56–0.92)	0.0096
	Model 3 ^4^	1.02 (0.89–1.16)	0.82	0.73 (0.57–0.93)	0.012
Acting with awareness	Model 1 ^2^	1.02 (0.94–1.10)	0.68	0.89 (0.77–1.03)	0.13
	Model 2 ^3^	1.04 (0.96–1.13)	0.33	0.90 (0.78–1.05)	0.17
	Model 3 ^4^	1.04 (0.96–1.13)	0.32	0.91 (0.78–1.05)	0.20
Describing	Model 1 ^2^	0.99 (0.92–1.07)	0.82	0.91 (0.80–1.05)	0.19
	Model 2 ^3^	1.01 (0.94–1.08)	0.84	0.91 (0.80–1.05)	0.19
	Model 3 ^4^	1.01 (0.94–1.09)	0.78	0.91 (0.80–1.05)	0.20
Observing	Model 1 ^2^	0.92 (0.85–0.99)	0.030	0.90 (0.76–1.06)	0.20
	Model 2 ^3^	0.95 (0.88–1.03)	0.19	0.90 (0.76–1.07)	0.24
	Model 3 ^4^	0.95 (0.89–1.04)	0.27	0.91 (0.77–1.07)	0.26
Non-judging	Model 1 ^2^	1.01 (0.94–1.09)	0.71	0.86 (0.75–0.99)	0.039
	Model 2 ^3^	1.01 (0.94–1.09)	0.74	0.86 (0.75–0.99)	0.037
	Model 3 ^4^	1.01 (0.94–1.09)	0.75	0.86 (0.75–0.99)	0.040
Non-reactivity	Model 1 ^2^	0.98 (0.89–1.07)	0.61	0.75 (0.63–0.92)	0.0040
	Model 2 ^3^	1.00 (0.91–1.10)	0.99	0.77 (0.64–0.93)	0.0063
	Model 3 ^4^	1.00 (0.91–1.10)	0.93	0.77 (0.64–0.94)	0.0081

Abbreviations: OR, Odds Ratio; 95% CI, 95% Confidence Interval; ^1^ Values ranged from 0 to 5 (higher scores indicated a greater mindfulness); ^2^ Model 1: adjusted for age, sex, education level, and familial status; ^3^ Model 2: Model 1 + physical activity and smoking status; ^4^ Model 3: Model 2 + energy intake and mPNNS-GS score.

**Table 3:** Multivariable logistic regression models between overall mindfulness ^1^, the metabolic syndrome and its components according to depressive symptomatology in 17,490 individuals (NutriNet-Santé Study, France, 2011–2014).

	Models	No Depressive Symptomatology *n* = 14,760		Depressive Symptomatology *n* = 2730	
		OR (95% CI)	*P*	OR (95% CI)	*P*
Elevated WC (≥94 cm for men. ≥80 cm for women)	Model 1 ^2^	0.85 (0.78–0.92)	<0.0001	0.77 (0.65–0.91)	0.0028
	Model 2 ^3^	0.89 (0.82–0.96)	0.0050	0.78 (0.66–0.93)	0.0064
	Model 3 ^4^	0.90 (0.83–0.98)	0.012	0.80 (0.67–0.96)	0.014
High blood pressure (SBP ≥ 130 and/or DBP ≥ 85 mm Hg) or treatment	Model 1 ^2^	1.01 (0.93–1.11)	0.78	0.86 (0.72–1.04)	0.11
	Model 2 ^3^	1.02 (0.93–1.11)	0.68	0.88 (0.73–1.05)	0.16
	Model 3 ^4^	1.03 (0.94–1.12)	0.53	0.88 (0.73–1.06)	0.18
High triglycerides (≥ 150 mg/dL) or treatment	Model 1 ^2^	0.98 (0.85–1.12)	0.74	0.84 (0.64–1.09)	0.19
	Model 2 ^3^	1.01 (0.88–1.16)	0.88	0.82 (0.63–1.07)	0.15
	Model 3 ^4^	1.01 (0.88–1.16)	0.91	0.83 (0.63–1.08)	0.16
Low HDL-cholesterol (<40 mg/dL for men. <50 mg/dL for women) or treatment	Model 1 ^2^	1.03 (0.90–1.18)	0.65	0.70 (0.54–0.92)	0.0094
	Model 2 ^3^	1.06 (0.92–1.21)	0.43	0.72 (0.55–0.94)	0.015
	Model 3 ^4^	1.05 (0.92–1.20)	0.47	0.73 (0.55–0.95)	0.019
High fasting blood glucose (≥100 mg/dL) or treatment	Model 1 ^2^	1.02 (0.90–1.15)	0.74	0.68 (0.53–0.87)	0.0026
	Model 2 ^3^	1.04 (0.92–1.17)	0.57	0.68 (0.53–0.88)	0.0032
	Model 3 ^4^	1.04 (0.92–1.18)	0.54	0.69 (0.54–0.89)	0.0045

Abbreviations: OR, Odds Ratio; 95% CI, 95% Confidence Interval; ^1^ The rage of values were from 0 to 5 (higher scores indicated a greater mindfulness); ^2^ Model 1: adjusted for age, sex, education level, and familial status; ^3^ Model 2: Model 1 + physical activity and smoking status; ^4^ Model 3: Model 2 + energy intake and mPNNS-GS score.

## References

[1] Alberti K.G.M.M., Eckel R.H., Grundy S.M., Zimmet P.Z., Cleeman J.I., Donato K.A., Fruchart J.-C., James W.P.T., Loria C.M., Smith S.C. (2009). Harmonizing the Metabolic Syndrome: A Joint Interim Statement of the International Diabetes Federation Task Force on Epidemiology and Prevention; National Heart, Lung, and Blood Institute; American Heart Association; World Heart Federation; International Atherosclerosis Society; and International Association for the Study of Obesity. Circulation.

[2] Ford E.S. (2005). Risks for all-cause mortality, cardiovascular disease, and diabetes associated with the metabolic syndrome. Diabetes Care.

[3] World Health Organization, World Health Organization (2009). Global Health Risks: Mortality and Burden of Disease Attributable to Selected Major Risks.

[4] Vernay M., Salanave B., De Peretti C., Druet C., Malon A., Deschamps V., Hercberg S., Castetbon K. (2013). Metabolic syndrome and socioeconomic status in France: the French nutrition and health survey (ENNS, 2006–2007). Int. J. Public Health.

[5] Chen C.-C., Li T.-C., Chang P.-C., Liu C.-S., Lin W.-Y., Wu M.-T., Li C.-I., Lai M.-M., Lin C.-C. (2008). Association among cigarette smoking, metabolic syndrome, and its individual components: the metabolic syndrome study in Taiwan. Metabolism.

[6] Edwardson C.L., Gorely T., Davies M.J., Gray L.J., Khunti K., Wilmot E.G., Yates T., Biddle S.J. (2012). Association of sedentary behaviour with metabolic syndrome: a meta-analysis. PLoS ONE.

[7] Lutsey P.L., Steffen L.M., Stevens J. (2008). Dietary intake and the development of the metabolic syndrome. Circulation.

[8] Mommersteeg P.M., Kupper N., Denollet J. (2010). Type D personality is associated with increased metabolic syndrome prevalence and an unhealthy lifestyle in a cross-sectional Dutch community sample. BMC Public Health.

[9] Sutin A.R., Costa P.T., Uda M., Ferrucci L., Schlessinger D., Terracciano A. (2010). Personality and metabolic syndrome. Age.

[10] Matthews K.A., Kuller L.H. (2002). The relationship between psychological risk attributes and the metabolic syndrome in healthy women: antecedent or consequence?. Metabolism.

[11] Kabat-Zinn J. (2003). Mindfulness-Based Interventions in Context: Past, Present, and Future. Clin. Psychol. Sci. Pract..

[12] Chiesa A., Serretti A. (2011). Mindfulness-Based Interventions for Chronic Pain: A Systematic Review of the Evidence. J. Altern. Complement. Med..

[13] Shennan C., Payne S., Fenlon D. (2011). What is the evidence for the use of mindfulness-based interventions in cancer care? A review. Psychooncology.

[14] Bowen S., Chawla N., Collins S.E., Witkiewitz K., Hsu S., Grow J., Clifasefi S., Garner M., Douglass A., Larimer M.E. (2009). Mindfulness-based relapse prevention for substance use disorders: A pilot efficacy trial. Subst. Abuse.

[15] Williams J.M.G., Alatiq Y., Crane C., Barnhofer T., Fennell M.J., Duggan D., Hepburn S., Goodwin G. (2008). Mindfulness-based cognitive therapy (MBCT) in bipolar disorder: Preliminary evaluation of immediate effects on between-episode functioning. J. Affect. Disord..

[16] Camilleri G.M., Méjean C., Bellisle F., Hercberg S., Péneau S. (2015). Association between Mindfulness and Weight Status in a General Population from the NutriNet-Santé Study. PLoS ONE.

[17] Younge J.O., Leening M.J.G., Tiemeier H., Franco O.H., Kiefte-de Jong J., Hofman A., Roos-Hesselink J.W., Hunink M.G.M. (2015). Association Between Mind-Body Practice and Cardiometabolic Risk Factors: The Rotterdam Study. Psychosom. Med..

[18] Anderson J.G., Taylor A.G. (2011). The Metabolic Syndrome and Mind-Body Therapies: A Systematic Review. J. Nutr. Metab..

[19] Murray C.J.L., Vos T., Lozano R., Naghavi M., Flaxman A.D., Michaud C., Ezzati M., Shibuya K., Salomon J.A., Abdalla S. (2012). Disability-adjusted life years (DALYs) for 291 diseases and injuries in 21 regions, 1990–2010: A systematic analysis for the Global Burden of Disease Study 2010. Lancet.

[20] Bajaj B., Robins R.W., Pande N. (2016). Mediating role of self-esteem on the relationship between mindfulness, anxiety, and depression. Personal. Individ. Differ..

[21] Park S.J., Roh S., Hwang J., Kim H.A., Kim S., Lee T.K., Kang S.H., Ha Y.J., Jang J.W., Park S. (2016). Association between depression and metabolic syndrome in korean women: Results from the korean national health and nutrition examination survey (2007–2013). J. Affect. Disord..

[22] Daskalopoulou M., George J., Walters K., Osborn D.P., Batty G.D., Stogiannis D., Rapsomaniki E., Pujades-Rodriguez M., Denaxas S., Udumyan R. (2016). Depression as a risk factor for the initial presentation of twelve cardiac, cerebrovascular, and peripheral arterial diseases: data linkage study of 1.9 million women and men. PLoS ONE.

[23] Hercberg S., Castetbon K., Czernichow S., Malon A., Mejean C., Kesse E., Touvier M., Galan P. (2010). The Nutrinet-Santé Study: A web-based prospective study on the relationship between nutrition and health and determinants of dietary patterns and nutritional status. BMC Public Health.

[24] Heeren A., Douilliez C., Peschard V., Debrauwere L., Philippot P. (2011). Cross-cultural validity of the Five Facets Mindfulness Questionnaire: Adaptation and validation in a French-speaking sample. Rev. Eur. Psychol. Appl. Eur. Rev. Appl. Psychol..

[25] Baer R.A., Smith G.T., Hopkins J., Krietemeyer J., Toney L. (2006). Using self-report assessment methods to explore facets of mindfulness. Assessment.

[26] World Health Organization (2011). Waist Circumference And Waist-Hip Ratio: Report of a WHO Expert Consultation, Geneva, 8–11 December 2008.

[27] Lassale C., Galan P., Julia C., Fezeu L., Hercberg S., Kesse-Guyot E. (2013). Association between adherence to nutritional guidelines, the metabolic syndrome and adiposity markers in a French adult general population. PLoS ONE.

[28] Gilbert D., Waltz J. (2010). Mindfulness and Health Behaviors. Mindfulness.

[29] Hagströmer M., Oja P., Sjöström M. (2006). The International Physical Activity Questionnaire (IPAQ): A study of concurrent and construct validity. Public Health Nutr..

[30] Le Moullec N., Deheeger M., Preziosi P., Monteiro P., Valeix P., Rolland-Cachera M., Potier de Courcy G., Christides J., Cherouvrier F., Galan P. (1996). Validation of the photo manual used for the collection of dietary data in the SU. VI. MAX. study. Cah. Nutr. Diét..

[31] Etude Nutrinet-Santé (2013). Table de Composition des Aliments de l’étude Nutrinet-Santé.

[32] Black A.E. (2000). Critical evaluation of energy intake using the Goldberg cut-off for energy intake: Basal metabolic rate. A practical guide to its calculation, use and limitations. Int. J. Obes..

[33] Malik V.S., Popkin B.M., Bray G.A., Després J.-P., Willett W.C., Hu F.B. (2010). Sugar-sweetened beverages and risk of metabolic syndrome and type 2 diabetes. Diabetes Care.

[34] Esmaillzadeh A., Kimiagar M., Mehrabi Y., Azadbakht L., Hu F.B., Willett W.C. (2006). Fruit and vegetable intakes, C-reactive protein, and the metabolic syndrome. Am. J. Clin. Nutr..

[35] Neufcourt L., Assmann K., Fezeu L., Touvier M., Graffouillère L., Shivappa N., Hébert J., Wirth M., Hercberg S., Galan P. (2015). Prospective association between the dietary inflammatory index and metabolic syndrome: Findings from the SU. VI. MAX study. Nutr. Metab. Cardiovasc. Dis..

[36] Jordan C.H., Wang W., Donatoni L., Meier B.P. (2014). Mindful eating: Trait and state mindfulness predict healthier eating behavior. Personal. Individ. Differ..

[37] Marchiori D., Papies E.K. (2014). A brief mindfulness intervention reduces unhealthy eating when hungry, but not the portion size effect. Appetite.

[38] Beshara M., Hutchinson A.D., Wilson C. (2013). Does mindfulness matter? Everyday mindfulness, mindful eating and self-reported serving size of energy dense foods among a sample of South Australian adults. Appetite.

[39] Hudson J.I., Lalonde J.K., Coit C.E., Tsuang M.T., McElroy S.L., Crow S.J., Bulik C.M., Hudson M.S., Yanovski J.A., Rosenthal N.R. (2010). Longitudinal study of the diagnosis of components of the metabolic syndrome in individuals with binge-eating disorder. Am. J. Clin. Nutr..

[40] O’Reilly G.A., Cook L., Spruijt-Metz D., Black D.S. (2014). Mindfulness-based interventions for obesity-related eating behaviours: a literature review. Obes. Rev..

[41] Donofry S.D., Roecklein K.A., Wildes J.E., Miller M.A., Erickson K.I. (2016). Alterations in emotion generation and regulation neurocircuitry in depression and eating disorders: A comparative review of structural and functional neuroimaging studies. Neurosci. Biobehav. Rev..

[42] Goodall K., Trejnowska A., Darling S. (2012). The relationship between dispositional mindfulness, attachment security and emotion regulation. Personal. Individ. Differ..

[43] Troop N.A., Treasure J.L. (1997). Psychosocial factors in the onset of eating disorders: Responses to life-events and difficulties. Br. J. Med. Psychol..

[44] Abramowitz J.S., Tolin D.F., Street G.P. (2001). Paradoxical effects of thought suppression: a meta-analysis of controlled studies. Clin. Psychol. Rev..

[45] Johnston L., Bulik C.M., Anstiss V. (1999). Suppressing thoughts about chocolate. Int. J. Eat. Disord..

[46] Strober M. (1984). Stressful life events associated with bulimia in anorexia nervosa. Empirical findings and theoretical speculations. Int. J. Eat. Disord..

[47] Anicha C.L., Ode S., Moeller S.K., Robinson M.D. (2012). Toward a cognitive view of trait mindfulness: Distinct cognitive skills predict its observing and nonreactivity facets. J. Pers..

[48] Loucks E.B., Britton W.B., Howe C.J., Eaton C.B., Buka S.L. (2015). Positive Associations of Dispositional Mindfulness with Cardiovascular Health: the New England Family Study. Int. J. Behav. Med..

[49] Loucks E.B., Gilman S.E., Britton W.B., Gutman R., Eaton C.B., Buka S.L. (2016). Associations of Mindfulness with Glucose Regulation and Diabetes. Am. J. Health Behav..

[50] Daubenmier J., Moran P.J., Kristeller J., Acree M., Bacchetti P., Kemeny M.E., Dallman M., Lustig R.H., Grunfeld C., Nixon D.F. (2016). Effects of a mindfulness-based weight loss intervention in adults with obesity: A randomized clinical trial: Mindfulness-Based Weight Loss for Obesity. Obesity.

[51] Momeni J., Omidi A., Raygan F., Akbari H. (2016). The effects of mindfulness-based stress reduction on cardiac patients’ blood pressure, perceived stress, and anger: a single-blind randomized controlled trial. J. Am. Soc. Hypertens. JASH.

[52] Skilton M.R., Moulin P., Terra J.-L., Bonnet F. (2007). Associations between anxiety, depression, and the metabolic syndrome. Biol. Psychiatry.

[53] Goldbacher E.M., Bromberger J., Matthews K.A. (2009). Lifetime History of Major Depression Predicts the Development of the Metabolic Syndrome in Middle-Aged Women. Psychosom. Med..

[54] Brown D.B., Bravo A.J., Roos C.R., Pearson M.R. (2015). Five facets of mindfulness and psychological health: evaluating a psychological model of the mechanisms of mindfulness. Mindfulness.

[55] Caspi A., Sugden K., Moffitt T.E., Taylor A., Craig I.W., Harrington H., McClay J., Mill J., Martin J., Braithwaite A. (2003). Influence of life stress on depression: moderation by a polymorphism in the 5-HTT gene. Science.

[56] Liu H., Song H., Tian R., Chen L., Zhang W., Qiang Y. (2015). Association between occupational psychological stress and metabolic syndrome. Zhonghua Lao Dong Wei Sheng Zhi Ye Bing Za Zhi Zhonghua Laodong Weisheng Zhiyebing Zazhi Chin. J. Ind. Hyg. Occup. Dis..

[57] Wingenfeld K., Kuehl L.K., Boeker A., Schultebraucks K., Ritter K., Hellmann-Regen J., Otte C., Spitzer C. (2017). Stress reactivity and its effects on subsequent food intake in depressed and healthy women with and without adverse childhood experiences. Psychoneuroendocrinology.

[58] Bohlmeijer E., ten Klooster P.M., Fledderus M., Veehof M., Baer R. (2011). Psychometric Properties of the Five Facet Mindfulness Questionnaire in Depressed Adults and Development of a Short Form. Assessment.

